# Patient-centred care attitudes and knowledge: a national study of BDS students in New Zealand

**DOI:** 10.1186/s12909-023-04496-7

**Published:** 2023-07-18

**Authors:** Guangzhao Guan, Li Mei, Chuting Yu, Yue Tan, Chengbing Han

**Affiliations:** 1 Department of Oral Diagnostic and Surgical Sciences, Faculty of Dentistry, University of Otago, Dunedin, New Zealand; 2 Department of Oral Sciences, Faculty of Dentistry, University of Otago, 310 Great King Street, Dunedin, 9016 New Zealand; 3 Department of Oral and Maxillofacial Surgery, Weifang People’s Hospital, Weifang, Shandong China

**Keywords:** Patient-centred care, Sharing, Caring, Patient-practitioner orientation scale

## Abstract

**Objective:**

The aim of this study was to investigate the attitudes and knowledge towards patient-centred care among Bachelor of Dental Surgery (BDS) students in New Zealand.

**Method:**

The study was a mixed methods cross-sectional national study of the BDS students in New Zealand. All 2021 BDS students at the New Zealand’s National Centre for Dentistry, New Zealand, were recruited in the study. The Patient-Practitioner Orientation Scale (PPOS) questionnaire was used to evaluate the dental students’ attitudes and knowledge of patient centred care. The students’ perspectives on the BDS curriculum regarding patient-centred care were also collected. ANOVA and the Student’s T-test were used to compare the difference among the BDS years, gender, and background.

**Results:**

A total of N = 277 (277/346; 80% response rate) students completed the study. Female students had higher scores than male students for sharing (difference = 0.19, 95% CI 0.04–0.34, P = 0.01), caring (difference = 0.15, 95% CI 0.01–0.29, P = 0.03) and PPOS (difference = 0.17, 95% CI 0.05–0.30, P < 0.01). Domestic students had higher scores than international students for caring (difference = 0.35, 95% CI 0.21–0.50, P < 0.01) and PPOS (difference = 0.22, 95% CI 0.08–0.35, P < 0.01). Three main themes of patient-centred care were extracted from the qualitative analysis of students’ perspectives: (1) Understanding of the concept of patient-centred care, (2) Perception of the importance of patient-centred care in dentistry, and (3) Perspective on the curriculum about patient-centred care.

**Conclusion:**

Most dental students favoured a patient-centred approach. According to dental students, the patient-centred care component of their education should be increased.

## Introduction

Patient-centred care is recognized as a foundation of quality patient care and a key component of the doctor-patient relationship [1]. It can be defined as giving care that respects and responds to each patient’s unique preferences, requirements, and values and making sure that patient values inform all clinical judgements [2, 3]. The attitude of patient-centred care is a practitioner consciously adopting the patient’s perspective through acknowledgement of the social factors, including the perspectives of individuals, families and communities involved in healthcare and allowing the patient to communicate their preferences [4]. It has been found that good communication between doctors and patients on treatment options help patients feel respected and their concerns addressed [5–7]. Clear communication facilitated by a patient-centred attitude enhances the doctor-patient relationship, improves patient satisfaction, compliance, and clinical outcomes [7]. The benefits of patient-centred care are well documented: increased delivery efficiency, decreased costs, improved equity in the uptake of service, better health literacy and self-care, increased satisfaction with care, improved relationships between patients and their care providers, and an improved ability to respond to health-care crises [8–10].

Despite the evidence and growing acceptance by global policymakers about the importance of patient-centred care [8], it has proved to be challenging to implement in many of the current healthcare systems [11]. There is no “one model” of patient-centred and integrated health services [8]. Multilevel barriers have prevented the development of patient-centred care attitudes among healthcare providers, causing them to tend towards ‘provider-dominant’ and ‘disease-centred’ in their practice orientation. Major determinants of these barriers are related to the structural and organizational features of the healthcare system. For example, a prominent reason why patient-centred care is not consistently provided is that providers lack the necessary attitudes and skills [12].

A healthcare professional’s future attitude and skill in patient-centred care can be influenced by the training program. A comprehensive educational course about patient-centred care would be beneficial for the BDS curriculum. In addition, the sharing and caring parts of the patient-centred care could be enhanced through role modeling and mentoring by the clinical supervisors and tutors. It has been demonstrated that the role modeling from clinical supervisors and tutors, the consistent support provided to students in fostering the patient-centredness attitude, as well as their personal experiences within the dental clinic could positively influence students to develop patient-centred clinical practice [14].

Understanding the current students’ attitudes and knowledge of patient-centred care is critical for dental education; establishing patient-centred skills in the dental curricula would help prospective clinicians deliver quality healthcare and build effective health systems. In an effort to increase the application of patient-centred approaches, students’ attitudes towards patient-centred care have been assessed in various healthcare professions, such as medicine, nursing, and physiotherapy. However, another study suggested that healthcare students had low attitudes towards patient-centred care [15]. Patient-centred care is similarly essential in dentistry, and is a critical component of multidisciplinary management; however, little is known about dental students’ attitudes and knowledge towards patient-centred care.

The aim of this study was to investigate the attitudes and knowledge of patient-centred care of the Bachelor of Dental Surgery (BDS) students.

## Methods

Ethical approval for the study was granted by The University of Otago Human Research Ethics Committee (D21/065). Informed consent was obtained before the study was conducted.

All BDS students were recruited in the study. The current BDS students included BDS2 (N = 73; the second-year students who are mainly trained in the simulation with no patient contact), BDS3 (N = 96; the third-year students who have patient contact in pairs), BDS4 (N = 76; the fourth-year students who have patient contact individually), BDS5 (N = 101; the fifth-year students whose majority of training is in clinical settings). Health Sciences First Year (equivalent to “BDS1”) is a foundation year for the university’s health professional programmes, such as dentistry, medicine, and pharmacy, etc. However, Health Sciences First Year students were excluded from this study as they had not been selected in the BDS programme.

The recruitment was carried out in the student common rooms, lecture theatre and simulation lab. The leaflets were used for recruitment. The participation was voluntary. Participants were asked to complete the Patient-Practitioner Orientation Scale (PPOS) questionnaire, which is a commonly used tool to assess patient-centred attitudes of students, providers, and patients, with a satisfactory internal consistency and validity [16]. A score higher than 3.5 indicates a patient-centred attitude [16, 17, 18, 19]. This PPOS questionnaire consists of 18 statements concerning the patient-provider relationship, in which participants are asked to what extent they agree or disagree with each of the statements. All statements were specifically written to reflect one of these two dimensions of patient-centredness previously identified, “Sharing” and “Caring”. Sharing items were written to reflect the degree to which clinicians should share power with the patient in terms of decision making and the extent to which information should be shared with the patient; Caring items were written to reflect the degree to which clinicians care about providing warmth and support to patients and the extent to which clinicians should go beyond the patient’s symptoms and care about the patient as a whole person [16].

In addition to the PPOS, data collection also included students’ knowledge, perception, and perspectives on BDS curriculum about the patient-centred care. All the open-ended questions were coded. Content analysis was used in this study.

### Statistics

Data were analyzed using the SPSS Statistics for Windows, Version 25.0. (Armonk, NY: IBM Corp). The comparisons across different BDS years, gender, and background were performed using one-way ANOVA and the Student’s T-test. A P-value of < 0.05 was considered to be of statistical significance.

## Results

A total of 277 BDS students completed the questionnaire, with an average response rate of 80.0% (277/346) (Table 1); the response rate of BDS2 was 97.2% (71/73), BDS3 was 77.0% (74/96), BDS4 was 68.4% (52/76), and BDS5 was 79.2% (80/101). Mean age of the students was 22.0 ± 2.8 years old, ranging from 18 to 35 years old. Female was 61.0% and male was 39.0%. 72.9% of the students were domestic and 27.1% were international.

**Table 1:** Demographic characteristics of the BDS (Bachelor of Dental Surgery) students included in the study (N, %)

Demographic	BDS 2	BDS 3	BDS 4	BDS 5	Total
**Gender**					
Female	44 (62.0%)	42 (56.8%)	35 (67.3%)	48 (60.0%)	169 (61.0%)
Male	27 (38.0%)	32 (43.2%)	17 (32.7%)	32 (40.0%)	108 (39.0%)
**Age**					
Mean ± Standard deviation	20.6 ± 3.2	21.9 ± 2.9	22.0 ± 2.0	23.4 ± 2.0	22.0 ± 2.8
**Ethnicity**					
NZ European	29 (40.8%)	13 (17.6%)	9 (17.3%)	12 (15.0%)	63 (22.7%)
Asian	9 (26.8%)	34 (45.9%)	30 (57.7%)	49 (61.3%)	132 (47.7%)
Maori and Pacific	8 (11.2%)	12 (16.2%)	10 (19.3%)	11 (13.8%)	41 (14.8%)
Other	15 (20.3%)	15 (20.3%)	3 (5.8%)	8 (10.0%)	41 (14.8%)
**Background**					
Domestic	58 (81.7%)	45 (60.8%)	42 (80.8%)	57 (71.3%)	202 (72.9%)
International	13 (18.3%)	29 (39.2%)	10 (19.2%)	23 (28.8%)	75 (27.1%)
**Total**	71 (100%)	74 (100%)	52 (100%)	80 (100%)	277 (100%)

### Attitudes towards patient-centred care

**Fig. 1:**
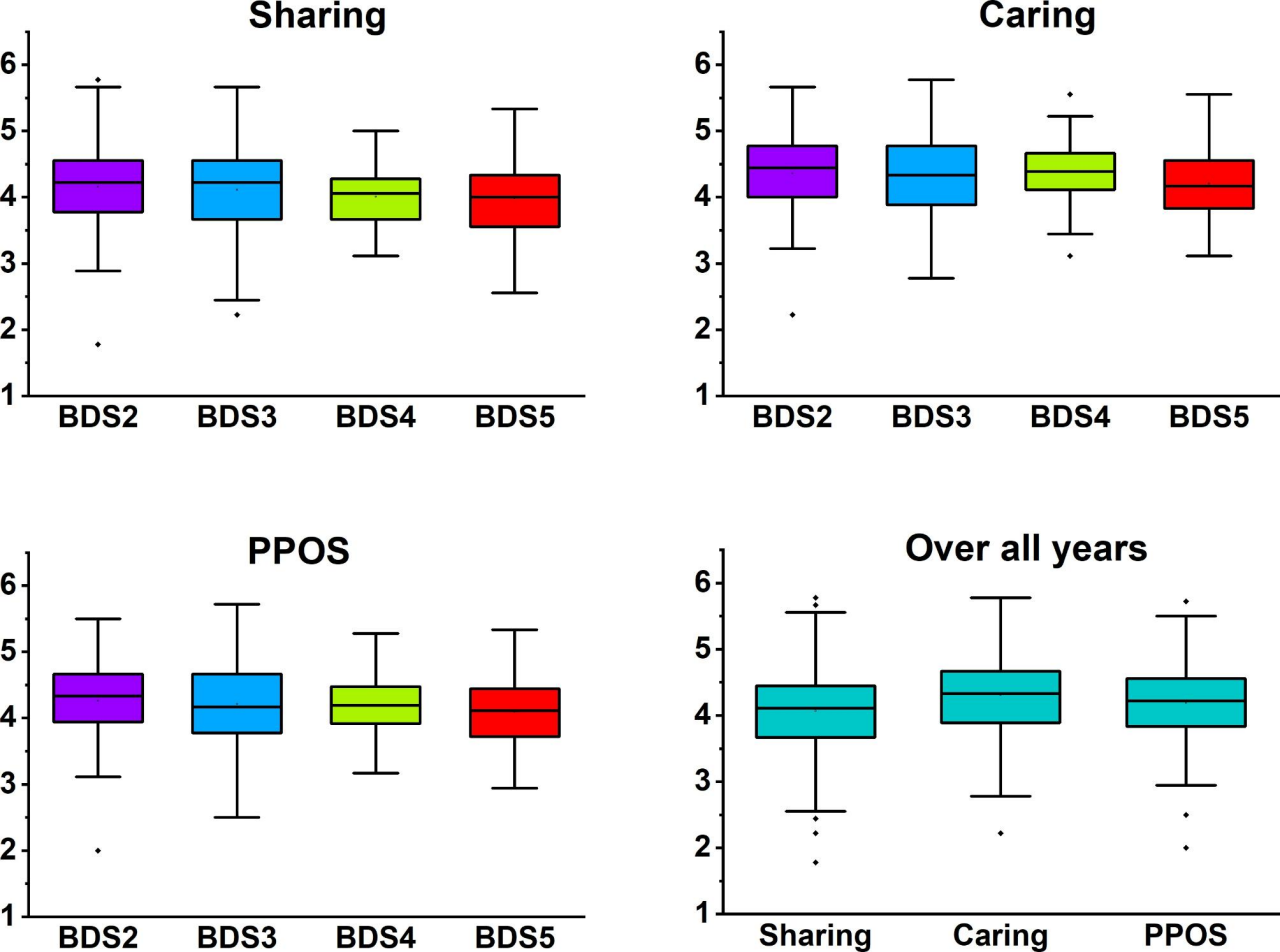
The Sharing caring and PPOS scores across all years

The average PPOS score of all students was 4.19 ± 0.52. Most students favoured a patient-centred approach in both sharing (4.07 ± 0.63) and caring (4.31 ± 0.57) categories (Fig. 1). The comparisons among different BDS years showed no significant differences in sharing (P = 0.33), caring (P = 0.23) or PPOS (P = 0.25) (Table 2).

**Table 2:** Comparisons of the scores (Mean ± SD) of sharing, caring and PPOS between years, gender and background

		Sharing		Caring		Patient-Practitioner Orientation Scale (PPOS)		
**Years**								
	BDS 2	4.15 ± 0.69	P = 0.33	4.36 ± 0.59	P = 0.23	4.26 ± 0.54	P = 0.25	
	BDS 3	4.11 ± 0.70		4.33 ± 0.60		4.22 ± 0.59		
	BDS 4	4.01 ± 0.46		4.38 ± 0.50		4.20 ± 0.41		
	BDS 5	3.98 ± 0.60		4.21 ± 0.56		4.10 ± 0.50		
**Gender**								
	Female	4.14 ± 0.59	**P = 0.01**	4.37 ± 0.53	**P = 0.03**	4.26 ± 0.47	**P < 0.01**	
	Male	3.95 ± 0.68	**95% CI 0.04–0.34**	4.22 ± 0.61	**95% CI 0.01–0.29**	4.09 ± 0.57	**95% CI 0.05–0.30**	
**Background**								
	Domestic	4.09 ± 0.61	P = 0.34	4.41 ± 0.53	**P < 0.01**	4.25 ± 0.50	**P < 0.01**	
	International	4.01 ± 0.68	95% CI -0.09–0.25	4.06 ± 0.59	**95% CI 0.21–0.50**	4.03 ± 0.57	**95% CI 0.08–0.35**	

The female students had higher scores than the male students for sharing (difference = 0.19, 95% CI 0.04–0.34, P = 0.01), caring (difference = 0.15, 95% CI 0.01–0.29, P = 0.03) and PPOS (difference = 0.17, 95% CI 0.05–0.30, P < 0.01). The domestic students had higher scores than the international students for caring (difference = 0.35, 95% CI 0.21–0.50, P < 0.01) and PPOS (0.22, 95% CI 0.08–0.35, P < 0.01). No significant difference of sharing was found between domestic and international students (difference = 0.08, 95% CI -0.09–0.25, P = 0.34) (Table 2).

### Understanding of the concept of patient-centred care

Patient-centred care is about managing a patient receiving care with dignity, respect and involving them in all decisions about their health. Most students had a reasonable understanding of patient-centred care which they learned from the BDS courses that was discussed not necessarily under the name of “patient-centred care”. Many of these comments recognized the values of patient-centred care and were associated with cultural competency.

*“…patient-centred care is the key to cultural competence…”- BDS2/F1*.

*“We should look more into cultural backgrounds and how we can better respect our patients who may be different to us.” - BDS3/M46*.

*“… dent school has good cultural education, for example Māori and Pacific….” - BDS4/F25*.

*“Role plays- whakawhanaungatanga.” - BDS5/F12* (Whakawhanaungatanga is a Māori word to describe the concept of establishing rapport and relationship with others. This is an important custom that is discussed in Māori cultural education that has been introduced to the healthcare education system to improve Māori patients’ access to dental healthcare. This aligns closely with multiple caring items in the PPOS questionnaire about understanding the patient’s background and appreciating that treatment is closely linked to collaborating with the patient’s self-care effort.)

### Perception of the importance of patient-centred in dentistry

All the BDS students in this study agreed that patient-centred care was significantly important in dental practices. There was no recorded comment of disagreement on the important role of patients in delivering quality healthcare. A consistent message from this study was that successful dental practices must place patients and their social well-being at the centre of decision making and understand factors spent outside the clinical settings, including patients’ behaviour, context and lifestyle.

*“… emphasis on patient-centred care is needed.” - BDS3/F26*.

*“A large focus right from the start that patient-centred care is very important.” - BDS4/F50*.

### Perspective on the BDS curriculum about patient-centred care

**Fig. 2:**
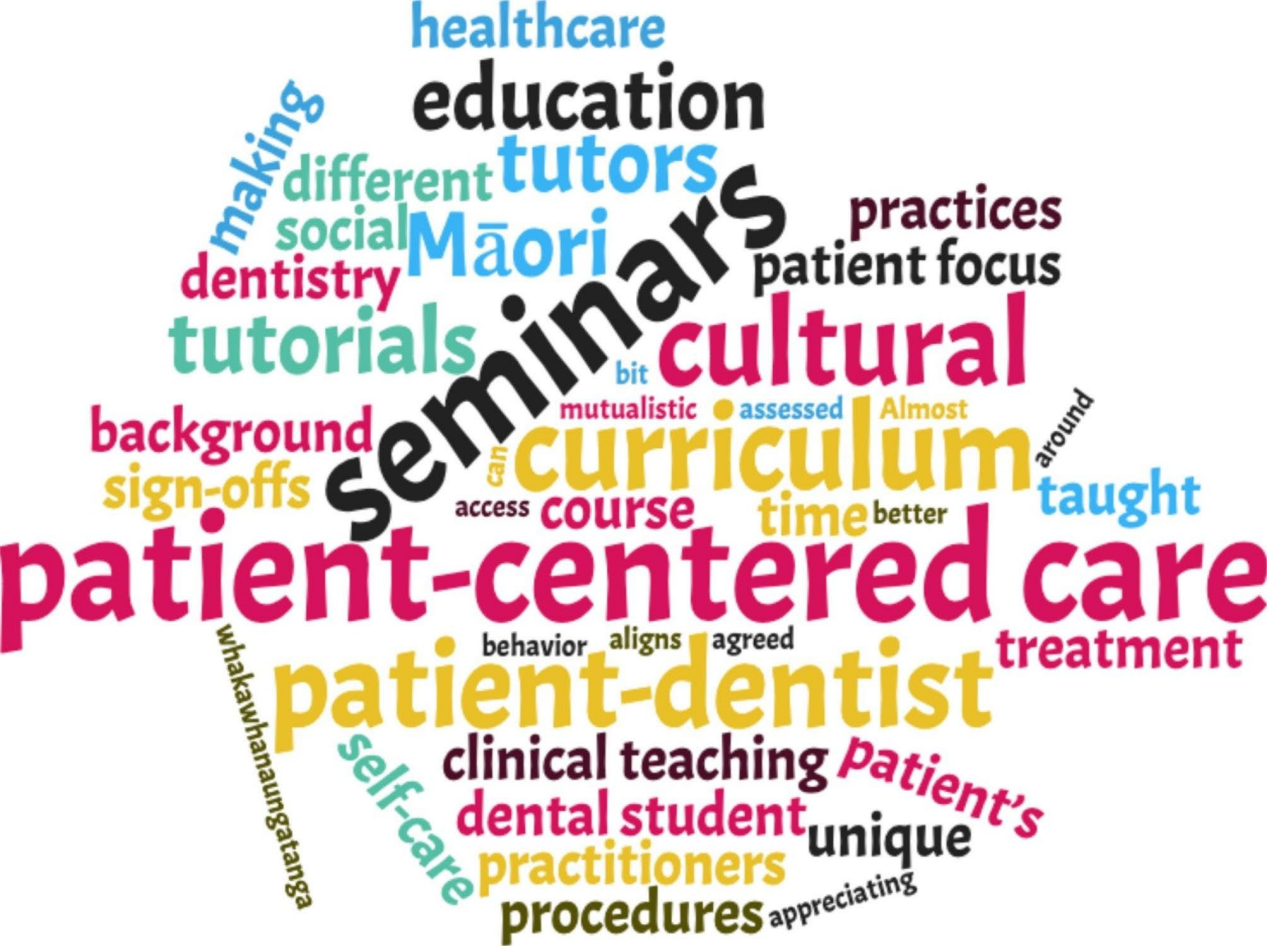
Word cloud created from the open-ended questions by NVivo 12

Figure 2 summarized students’ opinions on the current BDS curriculum about patient-centred care. Most students thought the present BDS programme should have more pertinent tutorials and training on patient-centred care. Students in the relative higher academic years would prefer greater instruction on practical procedures and communication skills for patient-centredness; students in the relative lower academic years appeared to have an expectation that they would receive courses or lectures on patient-centred care.

*“Need a bit more teaching around patient-centred care…”-BDS2/F16*.

*“Teach us how to interact with patient, know what info to be aware of before we get patients… more content regarding social interaction in a patient-dentist scenario”-BDS4/F3*.

*“It’s generally well taught through tutors, but the occasional tutor doesn’t realize that the student isn’t doing patient-centred care and rather coercing the patient into what they need for sign-offs.” - BDS5/F65* (Students are assessed through a checklist of competencies. There is a list of procedures that the curriculum expects a student to perform before graduation, also known as “sign-offs.“).

## Discussion

Patient-centred care is a universally recognized primary approach to high-quality healthcare amongst all health professions [20]. In the context of New Zealand culture, concepts that align with patient-centred care were sometimes discussed alongside with cultural education, as New Zealand is host to a wide variety of ethnic backgrounds. This study investigated dental students’ attitudes and knowledge towards patient-centred care, as well as their perspectives on the current dental curriculum regarding patient-centredness. The findings of the study suggested that most dental students, especially females and domestic students, favoured a patient-centred approach; and the students recommended an enhancement of patient-centred care component in their current BDS curriculum.

Students’ attitudes and knowledge towards patient-centred care have been extensively studied in medical settings; though patient-centred care is similarly essential in dentistry, dental students’ attitudes and knowledge towards patient-centred care is poorly understood. In this study, New Zealand’s dental students’ average PPOS score (4.19) appeared to be slightly higher than the medical students (3.38) in West Africa [21], the medical students (3.63) in China [22], the medical students (3.9) in Korea [23], and the speech & hearing sciences students (4.13) in India [24], the average score of chiropractic students in six countries (Canada, United States of America, United Kingdom, Denmark, France and Australia) (4.18) [25], but slightly lower than the medical students (4.57) in America [26] and the medical students (4.66) in Brazil [27]. Another study in the UK found that the mean PPOS was 4.0 for students in medicine, nursing, physiotherapy, and speech and language therapy [28]. This suggests that the dental students in New Zealand generally have a reasonable understanding and attitude towards the patient-centred care but also there is still space for further improvement, which is in agreement with a previous study in dentistry [29].

Practitioners’ demographic characteristics (genders and background) have been found to be associated with their attitudes towards patient-centred care [30]. In the study, female students had a relatively higher score of patient-centred care than the male students, which is in agreement with the previous studies [23, 27, 31, 32]. This may be due to the gender difference when it comes to communication. It has been found that females, compared with male practitioners, are relatively more engaged in positive discussion, question-asking, partnership-building, and information-giving in term of health, medical and psychosocial topics, and shared decision making [33, 34]. New Zealand domestic students had slightly higher overall PPOS and caring scores than the international students in this study, which might be related to the socio-cultural difference across different countries and different cultural backgrounds. It has been found that the patient-centred care attitude could be influenced by the religions, interpersonal attitude, and family background associated with healthcare system and education level [35, 36, 37]. A previous study has also found that a student’s experience of healthcare systems, either as a patient or having to care for the family, was associated with relatively higher PPOS scores [38].

It is uncertain whether student’s attitude towards patient-centred care changes during the course of academic years, but some studies have reported that the students’ attitudes in their later academic years seemed to be more doctor-centred or paternalistic than these among students during the early academic years [32, 39, 40]. In the literature, this was often attributed to the ‘hidden curriculum’ and inappropriate modeling by clinical tutors [14]. In this study, there seemed indeed a slight decreasing trend of sharing, caring and PPOS scores with the increasing academic years, though the difference among the academic years had no statistical significance. Patient-centred care emphasizes the understanding that the patient is an individual rather than an object of a disease entity and emphasizes upon the unique identity of every patient [13].

It has also been suggested that some continuing professional development course in patient-centred care for staff, supervisors, and tutors would be helpful for the education [1, 41]. The way teachers relate to students might influence how students relate to their patients; a supportive student-provider relationship and a student-centred education are important [13]. Based on the students’ perspectives in the study, the components of doctor-patient communication and soft skills related to patient-centred care in the current BDS curriculum could be enhanced; and the skills in sharing and gathering information, explanation, planning, and reaching consensus on problems could be promoted in the curriculum optimization. Case studies and role-plays may facilitate the practical acquisition of these skills, especially in areas like learning motivation [42].

There were a number of limitations in the study. Its involvement of dental students from a single centre may limit the global generalizability of the findings to other nations. Secondly, this study mainly investigated the views of BDS students, and their perspectives may differ from the undergraduate students majoring in the other dental degrees such as Bachelor or Oral Health, Bachelor of Dental Technology. It is also unknown whether the international students’ different culture backgrounds and language differences impacted their understanding and interpretation of the questionnaire of the study.

## Conclusion

Most dental students favored a patient-centered approach during their BDS study, especially the female and domestic students. According to dental students, the patient-centred care component of their education should be increased.

## Data Availability

All data have been presented in the article. The datasets used in the current study are available from the corresponding author on request.
